# Horner Syndrome After Radiofrequency Ablation of a Benign Thyroid Nodule

**DOI:** 10.7759/cureus.81227

**Published:** 2025-03-26

**Authors:** Nikita Mokhashi, Joseph Tobias, Peter Angelos, Hassan Shah

**Affiliations:** 1 Department of Ophthalmology and Visual Sciences, University of Chicago, Chicago, USA; 2 Department of Surgery, Endocrine Surgery Program, University of Chicago, Chicago, USA

**Keywords:** anisocoria, horner syndrome, radiofrequency ablation, surgery complication, thyroid nodule

## Abstract

A 56-year-old woman presented for evaluation of a right thyroid nodule causing dysphagia. Thyroid function tests were normal. Ultrasound showed an isoechoic right thyroid nodule measuring 3.6 × 2.1 × 3.5 cm. After fine needle biopsy demonstrated benign pathology, ultrasound-guided radiofrequency ablation (RFA) of the nodule was performed. One day after the procedure, the patient developed a miotic, non-reactive right pupil, right upper eyelid and cheek swelling, right upper eyelid ptosis, and injection of the right eye. The patient was diagnosed with Horner syndrome (HS). RFA is a safe, minimally invasive procedure for the treatment of benign thyroid nodules. However, providers performing RFA should be aware of the possibility of HS in the immediate post-procedural period and beyond.

## Introduction

Horner syndrome (HS), which presents with unilateral miosis, ptosis, and anhidrosis, occurs from damage to the sympathetic fibers along their course from the hypothalamus to the superior cervical ganglion to the postganglionic fibers that innervate the sweat glands and blood vessels of the face as well as the iris dilator and superior tarsal muscles [1]. HS is a known but very rare complication of thyroid surgery due to unintentional damage to the cervical sympathetic fibers that run deep to the internal carotid artery [2].

Thermal ablation techniques such as radiofrequency ablation (RFA) are increasingly used in the treatment of benign thyroid nodules and have been shown to be safe and effective alternatives to surgery, achieving volume reduction rates of up to 95% over time [3,4]. RFA is a minimally invasive procedure performed under direct sonographic visualization using high-frequency alternating currents to generate thermal energy to induce coagulative necrosis and tissue destruction [5]. Given the relative novelty of these techniques in North America, practitioners are still cataloguing the range of complications that result from the thermal ablation of benign thyroid nodules [3]. Although altogether rare, reported complications include transient or permanent injury to the recurrent laryngeal nerve leading to vocal cord paresis and hoarseness, transient thyroiditis, delayed nodule rupture, edema, and hematoma [6].

The sympathetic chain runs along the spine and posterior to the carotid sheath, which typically places it posterolateral to the normal anatomic location of the thyroid. This anatomic relationship places it at risk for damage during ablative thyroid procedures, especially in nodules with posterior extension. Herein, we describe a case of a transient HS after radiofrequency ablation of a 3.5-cm benign, non-functional, symptomatic thyroid nodule. To our knowledge, HS after RFA for a thyroid nodule has only been documented in one other case report to date [7].

## Case presentation

**Figure 1:**
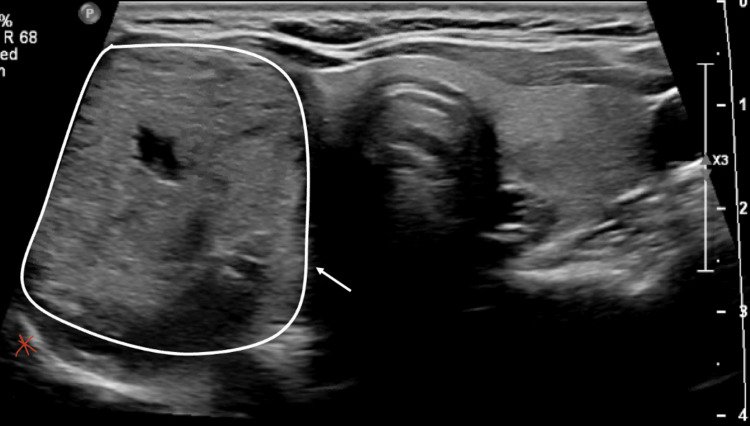
Ultrasound of the thyroid gland Ultrasound of the thyroid gland with thyroid nodule (white circle and arrow) with the typical location of the sympathetic chain indicated by red asterisk

A 56-year-old woman was referred to Endocrine Surgery for evaluation of a right thyroid nodule causing dysphagia. Thyroid function tests were normal. Ultrasound of the thyroid showed an isoechoic thyroid nodule within the right thyroid lobe measuring 3.6 × 2.1 × 3.5 cm (Figure 1). Two serial fine needle aspiration biopsies demonstrated benign pathology. A decision was made to proceed with ultrasound-guided RFA of the nodule using the STARmed Thyroid RFA system. Lidocaine (1%) was used as a local subcutaneous anesthetic. Under ultrasound guidance, a 22g spinal needle was also used to inject 1% lidocaine around the anterolateral part of the thyroid capsule. The nodule was targeted with an 18-gauge needle and 1-cm active tip length electrode with a power of 40 watts. Several passes were made under ultrasound guidance, and the tip was slowly withdrawn once the impedance reached 150 ohms using the standard trans-isthmic moving shot technique. Once 80% of the nodule was ablated, the electrode was removed, and the entry sites were compressed for five minutes. Once there was no evidence of hematoma, a dressing and ice were applied.

**Figure 2:**
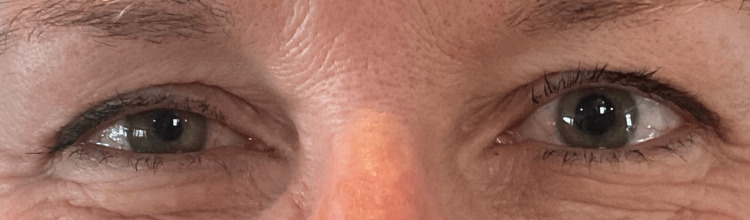
Eyes with anisocoria and right upper eyelid ptosis Anisocoria with the right pupil smaller than the left pupil and right upper eyelid ptosis suggestive of Horner syndrome from right sympathetic chain damage

One day after the procedure, the patient developed a miotic, non-reactive right pupil, right upper eyelid and cheek swelling, right upper eyelid ptosis, and injection of the right eye. She subsequently presented to the emergency room for these symptoms, where a magnetic resonance imaging (MRI) of the brain and magnetic resonance angiography (MRA) of the head and neck were performed. MRA of the head and neck did not have any sign of dissection, occlusion, or acute thrombosis of the vertebral arteries; common carotid arteries; internal and external carotid arteries; basilar artery; or anterior, middle, and posterior cerebral arteries. MRI of the brain was normal. The patient was evaluated by ophthalmology and diagnosed with HS. Ten days after RFA, the patient's ptosis and miosis were seen to be improving (Figure 2). All symptoms had resolved by one month. A thyroid ultrasound nine months after the procedure demonstrated a slight decrease in the size of the right thyroid nodule to 2.9 × 1.8 × 1.9 cm.

## Discussion

RFA of benign, non-functional thyroid nodules has been shown to result in a 47.4%-96.9% reduction in volume with extremely low rates of complications such as vocal hoarseness, recurrent laryngeal nerve injury, nodule rupture, needle track seeding, and hematoma [6,8]. HS from RFA to treat a thyroid nodule is uncommonly reported and has only been noted in one prior case in the literature to date [7]. HS can be caused by any compression or damage to the sympathetic pathway. The sympathetic system has input to the pupillary dilator muscle, which controls pupillary dilation, and the Muller muscle, which plays a role in eyelid elevation. Damage to the sympathetic system thus results in an ipsilateral smaller pupil due to dysfunctional dilation as well as ipsilateral ptosis [9]. Various mechanisms can potentially cause HS. First is the close posterolateral anatomic relationship of the sympathetic chain to the thyroid gland. Second is that a branch from the inferior thyroid artery has been noted to supply the sympathetic trunk [10] and could be damaged during ablation, leading to local ischemia of the sympathetic trunk. Lastly, another potential cause is post-ablation hematoma and inflammation leading to compressive damage of the sympathetic chain or the peri-carotid sympathetic fibers [10].

In a systematic review of 159 cases of HS, the most common inciting causes were procedures in the neck, chest, skull base, and paraspinal region. Tumors were the second most common cause, and a minority of cases resulted from carotid dissection. Thyroid surgery is a common culprit, including open surgery, endoscopy, and other surgical methods [2]. In 2023, the American Thyroid Association released its consensus statement on ablation techniques for benign thyroid nodules [3]. While chemical ablation has been used for decades, thermal ablation has more recently become widely adopted and is a highly effective technique for the non-surgical treatment of benign nodules that provides a valid alternative to conventional surgical management. RFA is ideal in patients who have a symptomatic thyroid nodule between 2 and 3 cm in size that has been proven to be benign on two separate fine needle aspiration biopsies. The technique has been shown to achieve volume reduction rates of 90% or greater at 36 months [3,4].

HS from thermal ablation of the thyroid is relatively uncommon. There are a few cases of HS following high-intensity focused ultrasound ablation [11] and microwave ablation [9,12] for benign thyroid nodules. RFA has been shown to be a rare cause of HS when used for tonsil reduction in three cases [13,14]. HS occurred more commonly in 103/185 patients after stereotactic RFA to treat hypothalamic hamartomas in a systematic review of 12 studies [15]. RFA, which is used to treat hyperthyroidism in cats and hyperparathyroidism in dogs, has also been shown to result in HS [16,17].

In a retrospective study of 875 patients who underwent RFA for benign thyroid nodules or recurrent thyroid cancer, there was only one patient who had transient HS in the immediate postoperative period [7]. In this case, the nodule was close to the middle cervical sympathetic ganglion, and the authors hypothesized that HS was due to thermal injury or hematoma [7]. To our knowledge, there are no other reports of HS after thyroid nodule RFA. Current guidelines recommend the use of a trans-isthmic approach with a moving shot technique, as we did in our case. The trans-isthmic approach refers to inserting the electrode at the midline of the neck with advancement laterally into the thyroid nodule to limit heat exposure to the recurrent laryngeal nerve [18]. The moving shot technique involves initial advancement of the tip to the most posterolateral portion of the nodule with subsequent ablation bit by bit with slow retraction of the needle medially and anteriorly along the insertion track [18]. In our case, the patient's nodule extended quite posteriorly, making this a higher risk case for sympathetic chain damage (Figure 1). As the sympathetic chain is typically located posterolateral to the thyroid gland, there was likely transmitted thermal injury posteriorly to the right cervical sympathetic chain. Given the resolution of symptoms in our patient, it is also possible that there was a component of hematoma and inflammation-related compression that resolved with time. The practitioner was also standing on the patient's right side, reaching across the patient to use a trans-isthmic technique, which may have led to inadequate needle control. Our institutional practice has since changed to stand on the contralateral side of the target nodule so that one does not have to reach across the patient in order to start at the midline of the neck. Inadequate operator experience could also contribute to a higher risk of HS after ablation. Interestingly, most cases of HS following thyroid surgery occur in the immediate postoperative period, but in our case, HS presented one day after the procedure.

When performing thyroid ablation, it is important to minimize the risk of injury to the sympathetic chain by reducing power in posterior ablations and avoiding deep penetration of the thyroid gland when possible. It may be prudent to monitor patients for HS even after the immediate post-procedural period. When concerned about possible HS, it is important to get prompt ophthalmologic evaluation to confirm the diagnosis and further workup.

## Conclusions

RFA is generally a safe, minimally invasive procedure that can be used to treat benign thyroid nodules, although HS can be a rare complication. To our knowledge, only one case similar to ours has been described in the literature. Providers performing RFA for benign thyroid nodules should be aware of the possibility of HS as a postoperative complication and monitor closely for HS in the immediate postoperative period and beyond.
